# Associations of Ultra-Processed and Unprocessed/Minimally Processed Food Consumption with Peripheral and Central Hemodynamics and Arterial Stiffness in Young Healthy Adults

**DOI:** 10.3390/nu12113229

**Published:** 2020-10-22

**Authors:** Katarina Smiljanec, Alexis U. Mbakwe, Macarena Ramos-Gonzalez, Christina Mesbah, Shannon L. Lennon

**Affiliations:** Department of Kinesiology and Applied Physiology, University of Delaware, 540 S College Avenue, Newark, DE 19713, USA; ksmilja@udel.edu (K.S.); ambakwe@udel.edu (A.U.M.); macramos@udel.edu (M.R.-G.); cmesbah@udel.edu (C.M.)

**Keywords:** ultra-processed food, unprocessed food, minimally processed food, NOVA classification, blood pressure, arterial stiffness, wave reflection, augmentation index

## Abstract

Consumption of ultra-processed food (UPF) replaces the intake of freshly prepared unprocessed/minimally processed food (MPF) and is positively associated with hypertension and cardiovascular disease (CVD). The objective of this observational study was to investigate the relation between (**1**) UPF and (**2**) MPF with peripheral and central blood pressure (BP), wave reflection, and arterial stiffness. Habitual dietary intake, ambulatory BP, augmentation index (AIx), and pulse wave velocity (PWV) were assessed in 40 normotensive young adults (15 M/25 W; 27 ± 1 y; body mass index 23.6 ± 0.5 kg/m^2^). UPF consumption was positively associated with overall and daytime peripheral systolic BP (B = 0.25, 95% confidence interval (CI) 0.03, 0.46, *p* = 0.029; B = 0.32, 95% CI 0.09, 0.56, *p* = 0.008, respectively), daytime diastolic BP (B = 0.18, 95% CI 0.01, 0.36, *p* = 0.049) and daytime peripheral pulse pressure (PP; B = 0.22, 95% CI 0.03, 0.41, *p* = 0.027). MPF consumption was inversely associated with daytime peripheral PP (B = −0.27, 95% CI −0.47, −0.07, *p* = 0.011), overall and daytime central systolic BP (B = −0.27, 95% CI −0.51, −0.02, *p* = 0.035; B = −0.31, 95% CI −0.58, −0.04, *p* = 0.024, respectively), and nighttime central PP (B = −0.10, 95% CI −0.19, −0.01, *p* = 0.042). Both UPF and MPF were not associated with AIx nor PWV. These data suggest avoidance of UPF and consumption of more MPF may reduce CVD risk factors.

## 1. Introduction

Recent findings from epidemiological studies indicate a positive association of ultra-processed food (UPF) consumption with all-cause mortality [1,2] and noncommunicable diseases including hypertension [3], cardiovascular disease (CVD) [4], dyslipidemia [5], obesity [6,7,8], metabolic syndrome [9], and cancer [10]. UPFs are defined as “formulations of ingredients, mostly of exclusive industrial use, that result from a series of industrial processes” [11]. They are manufactured with additives and ingredients that rarely have culinary use. For example, high-fructose corn syrup, hydrogenated oils, soy protein isolate, flavor enhancers, and dyes are often found on the ingredient list. UPF products have poor nutrient quality as they are usually high in salt, sugar, and fat, contain little or no whole foods, and are depleted of fiber, micronutrients, and bioactive components [4,12].

Per capita sales of UPF increased worldwide from 2000 to 2015 [13]. This trend is alarming as increased consumption of UPF [12,14,15,16] replaces meals freshly prepared from unprocessed/minimally processed food (MPF) [17,18]. In the United States (U.S.), UPF consumption contributes 58% of energy intake [19] which is more than UPF consumption in other countries such as Canada [18], the United Kingdom [14], France [16], Spain [2], Brazil [7], and Chile [15].

Approximately half of cardiovascular and cardiometabolic disease deaths are attributable to dietary factors [20,21], emphasizing the need to assess the impact of UPF intake on cardiovascular health. The relation between blood pressure (BP) and several nutrients abundant in UPF has been established. Indeed, intakes of salt, sugar-sweetened beverages, and diets rich in fat and sugar are associated with increased risk of hypertension [22,23,24,25]. To date, only two studies have specifically investigated the effects of UPF on cardiovascular health. In a large French cohort, UPF consumption was associated with greater risk of overall CVD, and specifically coronary heart and cerebrovascular disease [4]. This study demonstrated that a 10% increase in UPF intake corresponded to a 12% increase in CVD incidence, while a 10% increase in MPF intake reduced CVD incidence by 9%. In a large Spanish cohort, UPF consumption of 5 servings/day was associated with a 21% greater risk of hypertension incidence compared to consumption of 3 servings/day [3]. These cohort studies suggest that increases in UPF consumption may be linked to increases in CVD. 

It is not known whether UPF consumption is associated with a higher BP in healthy adults without CVD. Transition from an ideal BP to prehypertension and hypertension increases in midlife, emphasizing the need to maintain an ideal BP throughout young adulthood [26]. There is also no data on the association of UPF consumption and central pressures as opposed to peripheral pressure. This is important as increased central pressures are suggested to have a greater influence on CVD than peripheral pressures and can independently predict cardiovascular events [27]. In addition to BP, wave reflection and arterial stiffness are independent predictors of cardiovascular health outcomes [27,28]. Arterial stiffening, resulting from structural changes in large conduit arteries, can lead to a loss of arterial elasticity and an increase in systolic BP. An increase in augmentation index (AIx), a measure of wave reflection, increases aortic systolic pressure and left ventricular load [29].

Taking into consideration the high consumption of UPF and high prevalence of hypertension and CVD in the U.S. (46% and 48% in adults, respectively [20]), the objective of our study was to investigate the relation of UPF and MPF consumption with peripheral and central BP, wave reflection, and arterial stiffness in a group of healthy adults. We hypothesized that (**1**) UPF consumption would be associated with greater peripheral and central BP, wave reflection, and arterial stiffness, and (**2**) MPF consumption would be associated with lower peripheral and central BP, wave reflection, and arterial stiffness in this population. Our exploratory objective was to investigate the relation of UPF and MPF consumption with BP and vascular measures in men and women.

## 2. Materials and Methods

### 2.1. Study Population

In total, 40 healthy adults between the ages of 18−45 participated in this study. The study protocol and procedures were approved by the Institutional Review Board of the University of Delaware (ID# 1008199) and conform to the provisions of the Declaration of Helsinki. Subjects were recruited through flyers posted around the University of Delaware campus and surrounding community, as well as through online advertisements. Informed consent was obtained from all subjects prior to enrollment in the study.

### 2.2. Subject Screening

Subjects reported to the laboratory for a screening visit and completed a medical history form, a Global Physical Activity Questionnaire [30], and a menstrual cycle history form (women only). Anthropometrics were collected and resting BP was taken after ≥10 min of seated rest (GE Medical Systems, Dash 2000, Milwaukee, WI, USA). The average of 3 measurements was reported. Subjects with a history of CVD, hypertension, malignant cancer, diabetes mellitus, renal impairment, or use of heart or BP medications were excluded. Subjects with a body mass index (BMI) of 30 kg/m^2^ or greater and those who used tobacco products were also excluded. Finally, women were excluded if they were pregnant or breast-feeding. During this visit, a trained researcher instructed subjects on how to fill out a 3-day food record.

### 2.3. Testing Visit

This was a cross-sectional observational study and subjects came to the laboratory for a single testing visit. Subjects arrived at the laboratory after having fasted for at least 4 h, refrained from caffeine and alcohol for 12 h, and refrained from exercise for 24 h. A research nurse or a trained researcher performed a blood draw via venipuncture for a complete blood count and a basic metabolic panel. A total of 7 subjects did not wish to have their blood drawn due to discomfort, while a complete blood count and basic metabolic panel was not done for 5 subjects. After an appropriate rest period, subjects underwent testing for BP and vascular measures. All women were tested in the early follicular phase of the menstrual cycle.

### 2.4. Dietary Intake Assessment

Habitual dietary intake was assessed with a 3-day food record including 2 weekdays and 1 weekend day. Subjects were instructed to complete the food record prior to coming to the testing visit and were familiarized with a printout of 2 D food models to aid in estimating portion sizes (adapted from [31]). During the testing visit, a trained researcher reviewed food records with the subject. Nutrient intake was analyzed using the Nutrient Data Systems for Research (NDSR 2018 and 2019, University of Minnesota, Minneapolis, MN, USA).

### 2.5. NOVA Food Classification

Food items were categorized into 4 groups based on the degree of processing according to the NOVA system of food classification [11]: (1) MPF (fresh fruits and vegetables, mushrooms, grains and pasta, nuts and seeds, fresh and pasteurized milk, plain yogurt, eggs, fresh or frozen unprocessed meat, herbs and spices, coffee, tea), (2) culinary ingredients (starches and flours, sugars and syrups, honey, salt, plant oil, animal fat, vinegar, cream), (3) processed food (canned or bottled fruits, vegetables, and legumes, fruits in syrup, canned fish, roasted and/or salted/sugared nuts and seeds, nut butters, homemade bread, beer, wine, cider), and (4) UPF (breakfast cereals, packaged bread, flavored yogurt and dairy products, half and half, lactose-free milk, milk alternatives, packaged sliced, processed, and creamed cheese, processed meats, meat alternatives, packaged (instant) soups and noodles, pasta sauces, ready-to-eat frozen dishes, condiments, sweet or salty packaged snacks, ice cream, confectionery, sugar-sweetened beverages, hard liquor). Cheese and dried, cured, or smoked meats were included in the UPF category as they contain additives such as colors, preservatives, and stabilizers. Relative energy intake of foods from each category was expressed as the percentage of total energy intake.

### 2.6. Ambulatory Blood Pressure Monitoring (ABPM)

ABPM was performed as it has a better prognostic value of CVD outcomes than office BP measurements [32]. Subjects wore the ambulatory BP monitor on their nondominant arm for the 24 h leading up to the testing visit (Oscar 2 with SphygmoCor, SunTech Medical) [33]. BP was taken automatically every 20 min during daytime and every 30 min during nighttime. Both central and peripheral BP was measured, including systolic BP, diastolic BP, mean arterial pressure (MAP), pulse pressure (PP), and aortic pressure. Data were accepted if at least 75% of the readings were successful.

### 2.7. Vascular Measures

#### 2.7.1. Augmentation Index (AIx)

A central aortic pressure wave was synthesized from the measured brachial artery pressure wave with the SphygmoCor XCEL system (AtCor Medical, Sydney, Australia), which uses a transfer function and is FDA approved [34]. AIx was obtained from the synthesized central pressure wave and calculated as the ratio between augmented pressure and central PP, or AIx = (P_2_−P_1_)/(P_s_−P_d_), where P_1_ is first shoulder of systolic pressure, P_2_ is second shoulder of systolic, P_s_ is peak systolic pressure, and P_d_ is end-diastolic pressure. Measures were performed after a 20-minute supine rest. An average of 3 measures was reported.

#### 2.7.2. Pulse Wave Velocity (PWV)

Carotid-femoral PWV, a gold standard for assessing arterial stiffness [35], was measured using applanation tonometry and the Sphygmocor XCEL system (AtCor Medical, Sydney, Australia). Carotid and femoral pressure waveforms were recorded simultaneously using a high-fidelity strain-gauge transducer (Millar Instruments, Houston, TX, USA) placed over the carotid artery and a BP cuff placed on the upper thigh, respectively. PWV distance was measured using the subtraction method where proximal distance (carotid measurement site to the sternal notch) was subtracted from distal distance (sternal notch to the thigh cuff). Carotid-femoral PWV was calculated by dividing the measured aortic distance (distal–proximal) by the average measured time delay between the initial upstroke of the corresponding carotid and femoral waveforms. The measure was performed while the subject was in a supine position. The measure was performed once.

### 2.8. Blood Analysis

A fasting venous blood sample was taken for a complete blood count and basic metabolic panel assessment (Quest Diagnostics, Seacaucus, NJ, USA), and serum electrolytes (sodium, potassium, and chloride) (EasyElectrolyte Analyzer, Medica, Bedford, MA, USA).

### 2.9. Statistical Analysis

The primary outcomes were to assess the relation between (**1**) UPF consumption and central and peripheral BP, AIx, and PWV, and (**2**) MPF consumption and central and peripheral BP, AIx, and PWV. Data were assessed for normality, homoscedasticity, linearity, and absence of multicollinearity. Multiple linear regressions were performed with BMI, age, sex, and UPF or MPF as independent variables, and BP as an outcome variable. Multivariable analyses for AIx as an outcome variable were adjusted for heart rate, age, and sex, while height was included as an independent variable in a separate analysis. Analyses for PWV as an outcome variable were adjusted for MAP, BMI, and sex. In addition, we conducted exploratory analyses to investigate potential sex differences of UPF and MPF consumption on BP and vascular measures. When men and women were analyzed separately, BMI was used as the only covariate for analyses of BP variables, age, and heart rate for AIx, and MAP and BMI for PWV. Crude analyses included only UPF or MPF as an independent variable. Unstandardized beta coefficient was reported in multiple linear regression results. Pearson’s bivariate and partial (controlling for BMI) correlation was performed to assess correlations between UPF, MPF, macronutrients and micronutrients, as well as between UPF and MPF. An independent t-test was used to compare differences in nutrient intake between men and women, and between UPF and MPF. Statistical analyses were performed using SPSS (IBM SPSS, version 26.0, Chicago, IL, USA). Significance was set a priori at *p* < 0.05.

## 3. Results

### 3.1. Subjects

In total, 40 subjects completed the study. Table 1 lists demographic and biochemical parameters. Men and women were similar in age and were nonobese. Screening diastolic BP, hemoglobin, blood urea nitrogen, and creatinine were lower in women, while high-density lipoprotein was greater in women. However, all values were within normal limits. The 24 h ABPM and vascular health measures are listed in Table 2. The majority of BP variables were comparable between men and women with the exception of higher overall and nighttime heart rate, and higher nighttime aortic pressure in women. Nevertheless, all values were within normal limits.

### 3.2. Dietary Intake

Table 3 depicts habitual dietary intake as assessed by 3-day food records. As expected, men had greater energy intake than women, but there were no differences in normalized intake of carbohydrates, fat, sodium, potassium, phosphorus, calcium, magnesium, and alcohol. Men also consumed more protein than women. Relative energy contribution of UPF was 50.0 ± 2.4% of daily energy intake while relative energy contribution of MPF was 26.7 ± 2.1%. UPF consumption was inversely associated with MPF consumption (*r* = −0.71, *p* < 0.001). There were no differences in the relative energy contribution of each NOVA food category between sexes.

Pearson correlation revealed UPF consumption was negatively associated with potassium intake (*r* = –0.45, *p* = 0.004) and positively associated with added sugar intake (*r* = 0.40, *p* = 0.011). MPF consumption was positively associated with protein intake (*r* = 0.42, *p* = 0.007) and phosphorus (*r* = 0.35, *p* = 0.027). These relations persisted even after controlling for BMI and age (data not shown). We did not observe associations for UPF and MPF intake and other nutrients (carbohydrates, fat, saturated fat, sodium, calcium, magnesium), nor alcohol. We next looked at whether any relations existed between UPF and MPF, and normalized intake of the micronutrients. Normalized sodium, calcium, and added sugar intake from UPF was greater than intake from MPF (sodium: 742 ± 50 vs 310 ± 47 mg/day, *p* < 0.001; calcium: 268 ± 24 vs 104 ± 24 mg/day, *p* < 0.001; added sugar: 13 ± 2 vs 4 ± 1 g/day, *p* < 0.001) in all subjects. Normalized potassium intake from UPF was lower than from MPF (481 ± 50 vs 665 ± 59 mg/day, *p* < 0.020). There was no difference in normalized phosphorus and magnesium intake between UPF and MPF (phosphorus: UPF: 278 ± 23 mg/day, MPF: 236 ± 22 mg/day, *p* = 0.197; magnesium: UPF: 65 ± 6 mg/day, MPF: 70 ± 6 mg/day, *p* = 0.572). Normalized sodium, calcium, and added sugar intake from UPF was greater than intake from MPF in men and women, while there were no differences between phosphorus, magnesium, and potassium intake. Lastly, there were no differences in intake of mentioned micronutrients from UPF or MPF between men and women.

### 3.3. Ultra-Processed Food Consumption, BP, and Vascular Health

Table 4 contains results from multivariable analyses of the association of UPF and BP, AIx, and PWV adjusted for BMI, age, and sex. There were no significant observations following crude analyses. After adjustment, UPF consumption was positively associated with overall and daytime peripheral systolic BP (i.e., 1% increase in UPF intake was associated with 0.25 mmHg and 0.32 mmHg increase in overall and daytime peripheral systolic BP, respectively), daytime diastolic BP and daytime peripheral PP in all subjects. UPF consumption was also positively associated with daytime systolic BP, daytime diastolic BP, daytime peripheral PP, and daytime central systolic BP in women. There were no associations of UPF consumption and BP in men, however there was a trend for a positive association between UPF and daytime peripheral systolic BP. We did not observe any associations between UPF consumption and AIx and PWV.

### 3.4. Unprocessed/Minimally Processed Food Consumption, BP, and Vascular Health

Table 5 contains results from multivariable analyses of the association of MPF and BP, AIx, and PWV adjusted for BMI, age, and sex. After adjustment, MPF consumption was inversely associated with daytime peripheral PP in all subjects (1% increase in MPF consumption was associated with 0.27 mmHg reduction in daytime peripheral PP), which was also observed in crude analysis. In women, MPF consumption was inversely associated with laboratory peripheral systolic BP and the following ABPM BP components: daytime peripheral systolic BP, overall and daytime peripheral diastolic BP, overall MAP and daytime peripheral PP. All observations were significant prior to adjustment (data not shown).

We observed several associations of MPF consumption and central BP. After adjustment, MPF consumption was inversely associated with overall and daytime central systolic BP (i.e., 1% increase in MPF consumption was associated with 0.27 mmHg and 0.31 mmHg reduction in overall and daytime central systolic BP, respectively), and nighttime central PP in all subjects. These observations were not significant in crude analyses, however overall and daytime central systolic BP trended to be significant (data not shown). In women, we observed an inverse association of MPF consumption with overall, daytime, and nighttime central systolic BP, overall and daytime central diastolic BP, and overall and daytime aortic pressure. All observations were significant in crude analyses. Lastly, there was an inverse association between MPF consumption and AIx adjusted for heart rate in women, as well as when adjusted for heart rate and age, and heart rate and height (B = −0.37, 95% CI −0.73, −0.01, *p* = 0.046). There were no associations between MPF and peripheral nor central BP in men.

## 4. Discussion

The present study is the first to investigate the relation of UPF and MPF consumption with BP and vascular health in young healthy adults. The objective was to assess the relation between (**1**) UPF and (**2**) MPF with peripheral and central BP, arterial stiffness, and wave reflection. We partially confirmed our hypotheses as we demonstrated (**1**) positive associations of UPF consumption with peripheral BP, and (**2**) inverse associations of MPF consumption with peripheral and central BP. We found no associations between UPF and wave reflection, nor with UPF, MPF, and arterial stiffness. We also observed sex differences as UPF consumption was positively associated with peripheral and central BP in women, while MPF consumption was inversely associated with peripheral and central BP and AIx in women. We did not observe any associations in men.

Hypertension is a major risk factor for CVD that afflicts nearly half of the American population [20]. Therefore, maintaining an ideal BP throughout young adulthood is important as there is an increased risk for prehypertension and hypertension in midlife [26]. Even in the absence of a hypertension diagnosis at age 30, the lifetime risk of CVD is 46% [36]. Several modifiable lifestyle factors impact one’s risk for hypertension, including diet [20]. The American Heart Association encourages consumption of a heart-healthy diet (rich in fruits, vegetables, whole grains, fish and shellfish, nuts, seeds, and legumes, and low in saturated fat, sodium, sugar-sweetened beverages, and processed meat), as well as keeping BP (namely systolic) in a healthy range as important approaches for optimal cardiovascular health [20]. Peripheral systolic and diastolic BP are strongly associated with increased CVD risk and are often prioritized due to an abundance of evidence for their association with CVD and ease of assessment in clinical settings [37]. Importantly, central (aortic) pressures are physiologically more relevant to CVD development compared to peripheral pressures as vital organs (i.e., heart, kidneys, brain) are exposed to the load of central rather than peripheral pressures. However, central pressures are not routinely measured in clinical settings as more expensive equipment beyond auscultatory sphygmomanometers and automated BP monitors is required. In addition, both peripheral and central PP are more strongly related to vascular hypertrophy, atherosclerosis, and cardiovascular events than peripheral and central systolic BP [38].

We demonstrated several associations between UPF and MPF consumption with peripheral and central BP. A 10% increase in energy intake from UPF consumption with total energy intake held constant, was associated with a 2.5 mmHg and 3.2 mmHg increase in overall and daytime peripheral systolic BP, respectively, a 1.8 mmHg increase in daytime diastolic BP, and a 2.2 mmHg increase in daytime peripheral PP. In contrast, a 10% increase in energy intake from MPF consumption with total energy intake held constant, was associated with a 2.7 mmHg decrease in daytime peripheral PP, while the association was trending for the daytime peripheral systolic BP. The evidence for the association of MPF and central BP was compelling as we observed significant inverse relations between MPF intake and overall and daytime central systolic BP, and nighttime central PP. A 10% increase in energy intake from MPF was associated with a 2.7 and 3.1 mmHg decrease in overall and daytime central systolic BP and a 1.0 mmHg decrease in central PP. A decrease in central pressures would relieve the workload imposed on the left ventricle and coronary arteries. Our findings in both peripheral and central BP components emphasize the importance of making an effort to avoid UPF in one’s diet and consume more MPF.

We observed sex differences in our cohort, as associations with UPF and MPF consumption and BP were present in women, but not in men. These results indicate BP and vascular health of women may be more susceptible to the effects of dietary intake on BP and vascular health compared to men. These observations are in agreement with previous studies that show the association of UPF and excess weight and obesity are more pronounced in women than men [6,7]. There are metabolic and behavioral explanations as to why women could be more susceptible to the negative effects of UPF. Diets with a high glycemic index and glycemic load, which are attributes of UPF products [39], are associated with a greater risk of CVD in women than men [40]. Furthermore, stress-related consumption of highly processed food is more prevalent among women [41,42]. Socio-economic status may also impact hypertension risk in women more than in men [43]. Interestingly, evidence from a Spanish cohort demonstrated that subjects consuming the most UPFs (5 servings/day) were more likely to be less physically active men [3]. The annual probability of a transition from ideal BP to prehypertension with each consecutive year under 30 years is twice as high in men compared to women [26]. Hypertension is also more prevalent in men 20−45 years old than women, a gap that narrows with increasing age [20]. Furthermore, men generally consume a lower proportion of healthy foods and a higher proportion of unhealthy foods compared to women [44]. This is in contrast with our cohort as men were physically active and consumed a proportion of MPF and UPF comparable to women. Thus, our results supporting avoidance of UPF and selection of MPF are strengthened by having a sample of physically active men and women with comparable dietary patterns. It is important for future research to address these discrepancies.

To date, only one epidemiological study investigated the association of UPF consumption and hypertension [3]. In a large cohort study, 14,790 adults were followed for a mean of 9 years to assess hypertension incidence. Authors observed that UPF consumption of 5 servings/day was associated with a 21% greater risk of developing hypertension compared to UPF consumption of 3 servings/day. However, hypertension assessment was suboptimal as subjects self-reported their BP and office BP was validated in a subset of only 127 subjects. Similarly, habitual dietary intake was assessed via food frequency questionnaire for the previous year, a method that has low accuracy due to recall bias [45]. We used rigorous methodology to assess BP and food intake [32,37,46]. We assessed both peripheral and central BP via ABPM, a method recommended by the American College of Cardiology and American Heart Association [37], that has been shown to be a better predictor of CVD than office BP [32]. We also assessed habitual dietary intake via a 3-day food record that minimizes reliance on subjects’ memory as foods are recorded at the time of eating [46].

In the U.S., nearly two-thirds of energy intake come from UPF (58%) [19]. These data confirm prior results using similar food classifications to NOVA, which show 61% of food purchases in America are highly processed foods [47]. We and others [4] demonstrated that UPF and MPF consumption exhibit a strong inverse correlation. This would indicate that observed associations with BP in our study could be due to a concomitant decrease in MPF consumption and an increase in UPF consumption, or vice versa. Several models have been developed to investigate CVD mortality by replacing energy intake from UPF with MPF or processed food (foods made from MPF and culinary ingredients) [2,48,49]. Moreira and colleagues demonstrated that this would lead to a 10% reduction in CVD deaths by 2030 in the United Kingdom [48]. Similarly, replacing 25%, 50%, or 75% of energy from UPF with MPF and processed food would lead to a 5.5%, 11%, and 29% reduction in CVD deaths by 2030 in Brazil [49]. A team of researchers in Spain demonstrated that replacing 10% of energy from UPF with processed food would reduce total mortality by ~10%, while replacing the same proportion of energy with MPFs would further reduce it by ~55% [2]. Our study further supports these observations as we have demonstrated notable associations of energy intake from UPF and MPF with BP. These findings indicate that choosing MPF over UPF has immense potential for cardiovascular as well as overall health.

UPF products have lower levels of micronutrients compared to MPF [12]. Consequently, UPF intake is negatively associated with the intake of several micronutrients as the UPF content of these nutrients is at least twofold lower than in MPF (vitamins B12, vitamin D, vitamin E, niacin, pyridoxine, copper, iron, phosphorus, magnesium, selenium, zinc) [12]. Interestingly, UPF is positively associated with the intake of calcium, thiamin, and riboflavin [12], and we showed that calcium intake from UPF is greater than from MPF. These results are likely due to fortification of foods during manufacturing. UPF products are high in sodium, a micronutrient known for its direct relation with hypertension [22,23]. In our analyses, sodium intake was greater from UPF than from MPF. Furthermore, UPF consumption was negatively associated with potassium intake and intake of potassium was greater from MPF. Potassium has been recognized for its BP-lowering effects [50], and high potassium consumption is recognized in the Dietary Approaches to Stop Hypertension, a diet that results in a greater reduction of BP compared to other dietary patterns [50]. Consumption of UPF tends to promote an overconsumption of micronutrients that are less desirable for cardiovascular health.

The mechanisms behind the detrimental effects of UPF on cardiovascular physiology are not completely understood. Observations seen in our study could be due to a higher intake of sodium and lower intake of potassium from UPF, and vice versa (lower intake of sodium and higher intake of potassium from MPF). In our analyses, we did not adjust our model for the intake of those minerals due to a small sample size. However, in a large French cohort, Srour et al. [4] showed a positive association of UPF with incidence of CVD even after adjusting for various dietary factors, such as energy, fat, sugar, salt, and fiber content, consumption of sugary products, salty snacks, fats and sauces, red and processed meat, beverages, fruit, and vegetables. In the first randomized-controlled trial investigating the effects of UPF on energy intake, subjects consumed ~500 kcal/day more during the UPF diet compared to the MPF diet [51]. Diets were matched for energy, macronutrients, sodium, sugar, fat, and fiber. Furthermore, the difference in micronutrient intake from UPF and MPF was consistent between men and women as in our sample, indicating the micronutrient content of the foods may not be the mechanism for associations of UPF, MPF, and BP we have observed in women but not in men. These findings suggest other properties of UPF, components formed during processing, or even molecules in the packaging of UPF can interact with human physiology. Indeed, processing changes orosensory properties of UPF making it, for instance, softer and easier to chew, which then leads to increased eating rate, lower satiation, and excess energy intake [51,52]. Processing of whole foods is known to alter the original food matrix, i.e., the complex network of nutrients and nonnutrients. Nutrients exhibit different properties and function in whole foods compared to in isolation due to interactions with other nutrients, the food matrix, and the host metabolism [53]. In addition, artificial sweeteners and emulsifiers used in UPF formulations could negatively alter the gut microbiota composition and function as shown in animal models [54,55,56]. To date, there are no randomized controlled trials investigating the effect of UPF on the gut microbiota, however, a recent systematic review suggests ultra-processed very low-energy diets can both positively and negatively alter the gut microbiota composition [57]. Lastly, there is evidence from animal models and epidemiological studies in humans that molecules such as acrylamide, acrolein, bisphenol A, and additives commonly found in UPF may affect the cardiovascular system [54,58,59,60,61,62]. More research is needed to investigate the effects of various processing methods, additives, and their combined effects on human physiology.

Finally, the present study is not without limitations. One limitation is our small sample size and an imbalance in the distribution of men and women. We also cannot expand these findings to other races as only a fourth of our sample was nonwhite. Lastly, we did not include physical activity in our analyses to avoid overfitting the regression model and poor predictions. Larger cohorts are needed to establish associations between UPF, MPF, BP, and vascular health to confirm our findings.

## 5. Conclusions

In conclusion, we found compelling evidence for the association of UPF and MPF with peripheral and central BP in a cohort of young healthy adults, with limited associations for wave reflection and no associations for arterial stiffness. Maintaining ideal BP in young adulthood is immensely important as even a healthy 30-year-old has a one in two chance of developing CVD in their lifetime [36]. Hence, another viable approach for establishing healthy dietary patterns to prevent hypertension and CVD would be to avoid intake of UPF and choose more MPF. To put our findings in perspective, 10% of energy intake is ~200 kcal for an average person consuming 2000 kcal/day, an amount found in a 2 oz granola bar (UPF) or two medium bananas (MPF). As such, avoiding consumption of UPFs and choosing more MPFs would not require major changes in dietary habits and may have lifelong implications.

## Figures and Tables

**Table 1:** Subject characteristics.

	All Subjects	Men	Women	*p*-Value *
**Demographic data**				
N	40	15	25	
Ethnicity (H/NH)	3/37	2/13	1/24	
Race (W/B/A) ^a^	30/0/8	9/0/5	21/0/3	
Age (year)	27 ± 1	27 ± 1	27 ± 1	0.870
Height (cm)	168 ± 2	176 ± 2	164 ± 1	< 0.001
Mass (kg)	67 ± 2	76 ± 3	62 ± 1	< 0.001
BMI (kg/m^2^)	23.6 ± 0.5	24.2 ± 0.8	23.2 ± 0.5	0.296
Systolic BP, screening (mmHg)	111 ± 2	114 ± 2	109 ± 3	0.158
Diastolic BP, screening (mmHg)	70 ± 2	74 ± 2	67 ± 2	0.019
Heart rate, screening (bpm)	65 ± 2	62 ± 2	67 ± 2	0.099
Moderate physical activity (min/week) ^b,#^	360 ± 73	432 ± 171	312 ± 48	0.432
Vigorous physical activity (min/week) ^b^	91 ± 24	116 ± 47	74 ± 25	0.403
**Biochemical parameters**				
Hemoglobin (g/dL) ^c^	13.8 ± 0.2	14.5 ± 0.3	13.3 ± 0.3	0.010
Hematocrit (%) ^c^	43.5 ± 0.6	44.7 ± 1.2	42.7 ± 0.6	0.133
Serum sodium (mmol/L) ^d^	140.6 ± 0.6	140.8 ± 0.9	140.8 ± 0.9	0.677
Serum potassium (mmol/L) ^d^	4.0 ± 0.0	4.0 ± 0.0	3.9 ± 0.1	0.956
Serum chloride (mmol/L) ^d^	103.7 ± 0.5	103.5 ± 0.7	103.8 ± 0.7	0.791
Cholesterol, total (mg/dL) ^e^	161 ± 6	163 ± 9	158 ± 7	0.642
High density lipoprotein (mg/dL) ^e^	55 ± 2	50 ± 3	60 ± 2	0.019
Low density lipoprotein (mg/dL) ^e^	89 ± 5	96 ± 8	84 ± 6	0.235
Triglycerides (mg/dL) ^e^	82 ± 8	94 ± 16	72 ± 5	0.217
Fasting blood glucose (mg/dL) ^e^	88 ± 1	87 ± 2	89 ± 2	0.494
Blood urea nitrogen (mg/dL) ^e^	14 ± 1	16 ± 1	12 ± 1	0.022
Creatinine (mg/dL) ^e^	0.9 ± 0.0	1.0 ± 0.0	0.8 ± 0.0	< 0.001
Estimated glomerular filtration rate (mL/min) ^e^	100 ± 2	102 ± 3	98 ± 3	0.450

A, Asian; B, Black; BMI, body mass index; BP, blood pressure; H, Hispanic; NH, non-Hispanic; W, White. Values are means ± SEM. ^a^ Two subjects (1 man, 1 woman) did not report race. ^b^ N = 30. ^c^ N = 33. ^d^ N = 32. ^e^ N = 28. * Men vs. Women. ^#^ Includes physical activity at work, travel to/from places, and recreational physical activity.

**Table 2:** Ambulatory blood pressure and vascular data.

	All Subjects	Men	Women	*p*-Value *
**ABPM, peripheral BP**				
*Systolic BP (mmHg)*				
Overall	114 ± 2	117 ± 2	112 ± 2	0.149
Daytime	118 ± 2	121 ± 2	115 ± 3	0.090
Nighttime	103 ± 2	106 ± 3	100 ± 2	0.103
*Diastolic BP (mmHg)*				
Overall	67 ± 1	68 ± 1	67 ± 2	0.600
Daytime	70 ± 1	72 ± 2	70 ± 2	0.436
Nighttime	56 ± 1	58 ± 2	55 ± 2	0.271
*MAP (mmHg)*				
Overall	83 ± 1	84 ± 1	82 ± 2	0.368
Daytime	81 ± 2	79 ± 4	82 ± 3	0.559
Nighttime	72 ± 1	74 ± 2	70 ± 2	0.188
*Heart rate (bpm)*				
Overall	72 ± 1	66 ± 2	75 ± 2	0.002
Daytime	77 ± 2	76 ± 3	79 ± 2	0.390
Nighttime	63 ± 2	58 ± 2	68 ± 2	0.002
*Pulse pressure (mmHg)*				
Overall	47 ± 1	49 ± 1	45 ± 1	0.080
Daytime	49 ± 1	51 ± 2	47 ± 2	0.155
Nighttime	46 ± 1	48 ± 1	45 ± 1	0.157
**ABPM, central BP**				
*Systolic BP (mmHg)*				
Overall	104 ± 2	105 ± 2	103 ± 2	0.412
Daytime	106 ± 2	109 ± 2	105 ± 3	0.316
Nighttime	94 ± 2	96 ± 2	93 ± 2	0.390
*Diastolic BP (mmHg)*				
Overall	68 ± 1	69 ± 2	68 ± 2	0.614
Daytime	71 ± 2	72 ± 2	70 ± 2	0.564
Nighttime	57 ± 1	59 ± 2	56 ± 2	0.412
*Pulse pressure (mmHg)*				
Overall	36 ± 1	36 ± 1	35 ± 1	0.395
Daytime	35 ± 1	36 ± 1	35 ± 1	0.291
Nighttime	37 ± 1	37 ± 1	37 ± 1	0.742
*Aortic pressure (mmHg)*				
Overall	11 ± 1	10 ± 1	12 ± 1	0.156
Daytime	10 ± 1	10 ± 1	11 ± 1	0.362
Nighttime	12 ± 1	10 ± 1	13 ± 1	0.030
Nighttime systolic BP dip (%)	12 ± 1	12 ± 2	13 ± 2	0.799
Nighttime diastolic BP dip (%)	19 ± 2	18 ± 3	20 ± 2	0.606
**Wave reflection and arterial stiffness**				
Augmentation Index (%)	6.8 ± 1.8	4.6 ± 3.1	8.1 ± 2.1	0.338
Carotid-femoral pulse wave velocity (m/s)	5.5 ± 0.1	5.4 ± 0.1	5.6 ± 0.2	0.446

ABPM, ambulatory blood pressure monitoring; BP, blood pressure; MAP, mean arterial pressure. Values are means ± SEM. * Men vs. women.

**Table 3:** Habitual dietary intake from 3-day food records.

	All subjects	Men	Women	*p*-Value *
**NOVA Food Classification**				
Unprocessed/minimally processed (%)	26.7 ± 2.1	29.3 ± 4.4	25.2 ± 2.2	0.363
Culinary ingredients (%)	2.5 ± 0.6	1.3 ± 0.6	3.2 ± 0.8	0.062
Processed (%)	20.8 ± 1.6	20.7 ± 3.7	20.8 ± 1.5	0.967
Ultra-processed (%)	50.0 ± 2.4	48.8 ± 5.2	50.8 ± 2.4	0.728
**Nutrients**				
Energy intake (kcal/day)	2060 ± 83	2359 ± 167	1880 ± 69	0.004
Carbohydrates (%)	46 ± 1	46 ± 3	46 ± 1	0.880
Fiber (g/day)	11 ± 0	10 ± 1	11 ± 0	0.351
Added sugar (%)	10 ± 1	9 ± 1	11 ± 1	0.405
Protein (%)	17 ± 1	19 ± 1	16 ± 1	0.021
Fat (%)	36 ± 1	35 ± 2	37 ± 1	0.344
Saturated fat (%)	12 ± 1	11 ± 1	12 ± 1	0.295
Sodium (mg/day)	1486 ± 50	1494 ± 83	1481 ± 64	0.910
Potassium (mg/day)	1355 ± 51	1343 ± 94	1362 ± 61	0.856
Phosphorus (mg/day)	638 ± 18	661 ± 31	624 ± 21	0.322
Calcium (mg/day)	469 ± 27	470 ± 58	468 ± 26	0.982
Magnesium (mg/day)	166 ± 10	159 ± 16	171 ± 13	0.563
Alcohol (%)	0.3 ± 0.1	0.2 ± 0.1	0.4 ± 0.1	0.233

Note: Nutrient intake is normalized per 1000 kcal when expressed in mg or g. Food record from one male subject has 2 reported days instead of 3. Values are means ± SEM. * Men vs. women.

**Table 4:** Multivariable analyses of the association of ultra-processed food (UPF) consumption, blood pressure (BP), and vascular function, adjusted for age, sex and BMI.

	All Subjects		Men		Women	
	B (95% CI)	*p*-Value	B (95% CI)	*p*-Value	B (95% CI)	*p*-Value
Systolic BP, laboratory (mmHg)	0.05 (−0.17, 0.28)	0.641	−0.11 (−0.32, 0.09)	0.259	0.29 (−0.11, 0.68)	0.151
Diastolic BP, laboratory (mmHg)	0.01 (−0.19, 0.22)	0.892	−0.08 (−0.31, 0.16)	0.488	0.10 (−0.27, 0.47)	0.573
**ABPM, peripheral BP**						
*Systolic BP (mmHg)*						
Overall	**0.25 (0.03, 0.46)**	**0.029**	0.15 (−0.07, 0.38)	0.167	0.38 (−0.02, 0.78)	0.062
Daytime	**0.32 (0.09, 0.56)**	**0.008**	0.23 (−0.01, 0.47)	0.057	**0.49 (0.03, 0.95)**	**0.039**
Nighttime	0.20 (−0.04, 0.44)	0.096	0.18 (−0.13, 0.49)	0.221	0.22 (−0.26, 0.69)	0.349
*Diastolic BP (mmHg)*						
Overall	0.14 (−0.03, 0.31)	0.093	0.07 (−0.11, 0.26)	0.392	0.22 (−0.09, 0.54)	0.145
Daytime	**0.18 (0.01, 0.36)**	**0.049**	0.08 (−0.12, 0.27)	0.418	**0.36 (0.01, 0.71)**	**0.044**
Nighttime	0.11 (−0.08, 0.30)	0.240	0.08 (−0.19, 0.35)	0.510	0.11 (−0.26, 0.48)	0.549
*MAP (mmHg)*						
Overall	0.18 (−0.01, 0.36)	0.052	0.10 (−0.09, 0.28)	0.283	0.28 (−0.05, 0.61)	0.090
Daytime	−0.03 (−0.41, 0.36)	0.887	−0.01 (−0.57, 0.55)	0.974	−0.04 (−0.66, 0.59)	0.906
Nighttime	0.14 (−0.06, 0.33)	0.158	0.11 (−0.15, 0.38)	0.375	0.14 (−0.24, 0.52)	0.439
*Heart rate (bpm)*						
Overall	0.01 (−0.18, 0.20)	0.913	0.06 (−0.19, 0.31)	0.600	0.07 (−0.29, 0.43)	0.694
Daytime	0.17 (−0.06, 0.39)	0.147	0.20 (−0.14, 0.53)	0.218	0.05 (−0.24, 0.34)	0.724
Nighttime	−0.03 (−0.25, 0.19)	0.787	0.02 (−0.23, 0.26)	0.882	−0.14 (−0.57, 0.29)	0.498
*Pulse pressure (mmHg)*						
Overall	0.10 (−0.03, 0.23)	0.115	0.08 (−0.08, 0.23)	0.283	0.14 (−0.08, 0.36)	0.200
Daytime	**0.22 (0.03, 0.41)**	**0.027**	0.09 (−0.12, 0.30)	0.371	**0.46 (0.14, 0.78)**	**0.008**
Nighttime	0.10 (−0.04, 0.23)	0.170	0.10 (−0.07, 0.27)	0.240	0.11 (−0.16, 0.38)	0.391
**ABPM, central BP**						
*Systolic BP (mmHg)*						
Overall	0.23 (−0.01, 0.46)	0.060	0.05 (−0.17, 0.26)	0.629	0.39 (−0.01, 0.78)	0.054
Daytime	0.20 (−0.02, 0.41)	0.074	0.06 (−0.15, 0.28)	0.521	**0.56 (0.08, 1.04)**	**0.025**
Nighttime	0.13 (−0.08, 0.34)	0.215	0.07 (−0.16, 0.30)	0.512	0.23 (−0.25, 0.71)	0.329
*Diastolic BP (mmHg)*						
Overall	0.12 (−0.08, 0.33)	0.293	0.03 (−0.16, 0.22)	0.712	0.26 (−0.13, 0.64)	0.176
Daytime	0.16 (−0.05, 0.38)	0.134	0.06 (−0.15, 0.27)	0.556	0.38 (−0.08, 0.83)	0.098
Nighttime	0.09 (−0.10, 0.28)	0.326	0.06 (−0.16, 0.27)	0.562	0.13 (−0.31, 0.58)	0.535
*Pulse pressure (mmHg)*						
Overall	0.06 (−0.03, 0.16)	0.192	0.02 (−0.09, 0.13)	0.733	0.14 (−0.03, 0.31)	0.099
Daytime	0.7 (−0.04, 0.18)	0.215	0.02 (−0.11, 0.15)	0.743	0.17 (−0.04, 0.38)	0.099
Nighttime	0.04 (−0.04, 0.13)	0.330	0.01 (−0.08, 0.11)	0.744	0.12 (−0.06, 0.29)	0.183
*Aortic pressure (mmHg)*						
Overall	−0.01 (−0.10, 0.07)	0.723	−0.06 (−0.18, 0.06)	0.286	−0.00 (−0.16, 0.16)	0.997
Daytime	−0.01 (−0.09, 0.09)	0.983	−0.05 (−0.19, 0.08)	0.384	0.08 (−0.12, 0.27)	0.443
Nighttime	−0.05 (−0.13, 0.04)	0.265	−0.07 (−0.15, 0.01)	0.097	−0.06 (−0.27, 0.15)	0.549
Nighttime SBP dip (%)	−0.02 (−0.19, 0.16)	0.836	−0.06 (−0.30, 0.19)	0.624	−0.01 (−0.30, 0.27)	0.938
Nighttime DBP dip (%)	0.02 (−0.22, 0.27)	0.846	−0.05 (−0.44, 0.335)	0.776	0.11 (−0.26, 0.48)	0.537
**Wave reflection and arterial stiffness**						
*Augmentation Index (%)*						
Adjusted for HR	−0.13 (−0.34, 0.07)	0.199	−0.26 (−0.53, 0.005)	0.054	0.07 (−0.27, 0.42)	0.676
Adjusted for sex, age, HR ^a^	−0.07 (−0.25, 0.12)	0.482	−0.20 (−0.47, 0.07)	0.126	0.15 (−0.13, 0.43)	0.288
*Carotid-femoral PWV (m/s)*						
Adjusted for MAP	−0.01 (−0.02, 0.01)	0.272	−0.01 (−0.02, 0.007)	0.243	−0.01 (−0.04, 0.01)	0.238
Adjusted for sex, BMI, MAP ^b^	−0.01 (−0.02, 0.00)	0.146	−0.01 (−0.03, 0.007)	0.243	−0.02 (−0.04, 0.01)	0.180

^a^ Adjusted for age and HR in men and women. ^b^ Adjusted for BMI and MAP in men and women. ABPM, ambulatory blood pressure monitoring; B, unstandardized beta coefficient; BMI, body mass index; BP, blood pressure; CI, confidence interval; DBP, diastolic blood pressure; HR, heart rate; MAP, mean arterial pressure; PWV, pulse wave velocity; SBP, systolic blood pressure. Significant results are in bold.

**Table 5:** Multivariable analyses of the association of unprocessed/minimally processed food (MPF) consumption and blood pressure (BP) and vascular function, adjusted for sex, age, and BMI.

	All Subjects		Men		Women	
	B (95% CI)	*p*-Value	B (95% CI)	*p*-Value	B (95% CI)	*p*-Value
Systolic BP, laboratory (mmHg)	−0.10 (−0.34, 0.14)	0.405	0.14 (−0.08, 0.36)	0.191	**−0.47 (−0.86, −0.08)**	**0.021**
Diastolic BP, laboratory (mmHg)	−0.03 (−0.26, 0.19)	0.760	0.14 (−0.18, 0.38)	0.245	−0.32 (−0.69, 0.05)	0.088
**ABPM, peripheral BP**						
*Systolic BP (mmHg)*						
Overall	−0.19 (−0.44, 0.06)	0.123	−0.06 (−0.32, 0.21)	0.643	−0.44 (−0.90, 0.02)	0.062
Daytime	−0.26 (−0.53, 0.01)	0.055	−0.11 (−0.41, 0.20)	0.461	**−0.53 (−1.02, −0.05)**	**0.034**
Nighttime	−0.14 (−0.41, 0.13)	0.297	−0.10 (−0.46, 0.25)	0.540	−0.27 (−0.77, 0.24)	0.278
*Diastolic BP (mmHg)*						
Overall	−0.14 (−0.33, 0.04)	0.127	−0.03 (−0.24, 0.17)	0.737	**−0.37 (−0.71, −0.02)**	**0.033**
Daytime	−0.17 (−0.37, 0.02)	0.082	−0.03 (−0.25, 0.19)	0.765	**−0.46 (−0.81, −0.11)**	**0.013**
Nighttime	−0.08 (−0.30, 0.12)	0.402	−0.04 (−0.34, 0.27)	0.800	−0.27 (−0.65, 0.10)	0.142
*MAP (mmHg)*						
Overall	−0.16 (−0.36, 0.04)	0.123	−0.04 (−0.25, 0.18)	0.720	**−0.39 (−0.76, −0.02)**	**0.038**
Daytime	0.06 (−0.35, 0.48)	0.764	−0.05 (−0.56, 0.66)	0.860	−0.01 (−0.65, 0.67)	0.972
Nighttime	−0.10 (−0.32, 0.11)	0.337	−0.05 (−0.35, 0.25)	0.704	−0.28 (−0.66, 0.11)	0.150
*Heart rate (bpm)*						
Overall	−0.01 (−0.22, 0.20)	0.869	−0.02 (−0.29, 0.26)	0.902	−0.01 (−0.38, 0.37)	0.966
Daytime	−0.08 (−0.33, 0.17)	0.523	−0.05 (−0.44, 0.34)	0.801	−0.11 (−0.50, 0.27)	0.543
Nighttime	0.04 (−0.20, 0.27)	0.731	0.07 (−0.20, 0.33)	0.599	−0.00 (−0.47, 0.47)	0.995
*Pulse pressure (mmHg)*						
Overall	−0.06 (−0.20, 0.09)	0.427	−0.04 (−0.21, 0.14)	0.673	−0.06 (−0.32, 0.21)	0.647
Daytime	**−0.27 (−0.47, −0.07)**	**0.011**	−0.13 (−0.35, 0.10)	0.245	**−0.45 (−0.80, −0.10)**	**0.015**
Nighttime	−0.06 (−0.21, 0.09)	0.430	−0.07 (−0.26, 0.13)	0.448	−0.00 (−0.30, 0.30)	0.988
**ABPM, central BP**						
*Systolic BP (mmHg)*						
Overall	**−0.27 (−0.51, −0.02)**	**0.035**	−0.09 (−0.34, 0.15)	0.415	**−0.62 (−1.05, −0.18)**	**0.008**
Daytime	**−0.31 (−0.58, −0.04)**	**0.024**	−0.10 (−0.34, 0.15)	0.404	**−0.78 (−1.24, −0.32)**	**0.002**
Nighttime	−0.22 (−0.45, 0.01)	0.058	−0.15 (−0.40, 0.10)	0.220	**−0.48 (−0.93, −0.02)**	**0.041**
*Diastolic BP (mmHg)*						
Overall	−0.17 (−0.41, 0.07)	0.151	−0.04 (−0.26, 0.19)	0.732	**−0.46 (−0.88, −0.03)**	**0.036**
Daytime	−0.19 (−0.44, 0.06)	0.133	−0.03 (−0.28, 0.22)	0.812	**−0.55 (−1.01, −0.10)**	**0.020**
Nighttime	−0.14 (−0.36, 0.08)	0.199	−0.10 (−0.35, 0.14)	0.365	−0.34 (−0.78, 0.10)	0.121
*Pulse pressure (mmHg)*						
Overall	−0.11 (−0.21, −0.01)	0.058	−0.06 (−0.18, 0.07)	0.311	−0.18 (−0.38, 0.02)	0.078
Daytime	−0.11 (−0.24, −0.01)	0.070	−0.07 (−0.21, 0.08)	0.330	−0.20 (−0.43, 0.02)	0.076
Nighttime	**−0.10 (−0.19, −0.01)**	**0.042**	−0.06 (−0.16, 0.05)	0.252	−0.14 (−0.32, 0.05)	0.137
*Aortic pressure (mmHg)*						
Overall	−0.06 (−0.16, 0.03)	0.165	−0.01 (−0.17, 0.14)	0.838	**−0.21 (−0.37, −0.05)**	**0.015**
Daytime	−0.08 (−0.18, 0.04)	0.110	−0.02 (−0.18, 0.15)	0.829	**−0.29 (−0.45, −0.13)**	**0.001**
Nighttime	−0.04 (−0.13, 0.06)	0.465	0.00 (−0.11, 0.11)	0.989	−0.17 (−0.37, 0.03)	0.085
Nighttime SBP dip (%)	0.02 (−0.17, 0.22)	0.802	0.06 (−0.20, 0.32)	0.620	0.01 (−0.30, 0.33)	0.943
Nighttime DBP dip (%)	−0.03 (−0.29, 0.24)	0.852	0.02 (−0.39, 0.44)	0.906	0.01 (−0.41, 0.41)	0.989
**Wave reflection and arterial stiffness**						
*Augmentation Index (%)*						
Adjusted for HR	−0.05 (−0.30, 0.19)	0.657	0.18 (−0.17, 0.53)	0.284	**−0.36 (−0.71, −0.02)**	**0.042**
Adjusted for sex, age, HR ^a^	−0.03 (−0.24, 0.18)	0.777	0.18 (−0.13, 0.49)	0.237	**−0.31 (−0.60, −0.03)**	**0.034**
*Carotid-femoral PWV (m/s)*						
Adjusted for MAP	0.00 (−0.01, 0.02)	0.646	0.01 (−0.01, 0.03)	0.342	0.01 (−0.02, 0.04)	0.544
Adjusted for sex, BMI, MAP ^b^	0.01 (−0.01, 0.02)	0.481	0.01 (−0.01, 0.03)	0.364	0.01 (−0.02, 0.04)	0.519

^a^ Adjusted for age and HR in men and women. ^b^ Adjusted for BMI and MAP in men and women. ABPM, ambulatory blood pressure monitoring; B, unstandardized beta coefficient; BMI, body mass index; BP, blood pressure; CI, confidence interval; DBP, diastolic blood pressure; HR, heart rate; MAP, mean arterial pressure; PWV, pulse wave velocity; SBP, systolic blood pressure. Significant results are in bold.
